# Harnessing non-equilibrium hot electrons in a quantum-engineered ternary heterostructure for sub-ppb C9 biomarker detection

**DOI:** 10.1039/d5sc05009j

**Published:** 2025-08-18

**Authors:** Xiao Li, Wenyuan Zhao, Jiaxing Liu, Rong Li, Sitong Jia, Cancan Li, Deyu Bao, Shan Zhu, Peng Wang, Lei Huang, Songqiu Yang, Miao Yu, Xiang Liu, Zhenjie Xue, Tie Wang

**Affiliations:** a Tianjin Key Laboratory of Life and Health Detection, Life and Health Intelligent Research Institute, Tianjin University of Technology Tianjin 300384 P. R. China life@tjut.edu.cn wangtie@email.tjut.edu.cn; b Dalian Institute of Chemical Physics, Chinese Academy of Sciences P. R. China sqyang@dicp.ac.cn

## Abstract

Photoactivated sensors offer a safe, low-power alternative to thermal sensors, yet their performance against trace concentrations of weakly reactive biomarkers is fundamentally crippled by the rapid energy loss of photogenerated carriers *via* electron relaxation into the band-edge. This process limits the number of electrons available for sensing. Here, we overcome this limitation by introducing a new principle: non-equilibrium hot-electron-mediated chemoresistance. Our quantum-engineered CdSe@CdS-Au ternary heterostructure is expressly designed to win the kinetic race against relaxation. Compared with two-tip gold domains governed by cooled electron transfer, the multi-site specific Au structure enhances electron transfer rates by 86-fold to 1.60 × 10^12^ s^−1^ with efficiencies of 99%, indicating ballistic hot-electron injection from the semiconductor into multi-site Au nanodomains prior to relaxation, as verified by femtosecond transient absorption spectroscopy. Functionalization with 4-bromobenzenethiol enables selective detection of *trans*-2-nonenal at 0.70 ppb—a new benchmark in optical sensors and chemoresistive sensors. Furthermore, a portable six-channel UV-activated sensor chip based on this principle demonstrates a 97.9% diagnostic accuracy in simulated exhaled breath, showcasing a transformative pathway toward non-invasive screening of non-small cell lung cancer.

## Abstract 2

We develop a photoactivated hot-electron chemoresistive sensor, enabling a novel on-chip platform for sub-ppb detection of C9 biomarkers at room temperature.

## Graphical abstract

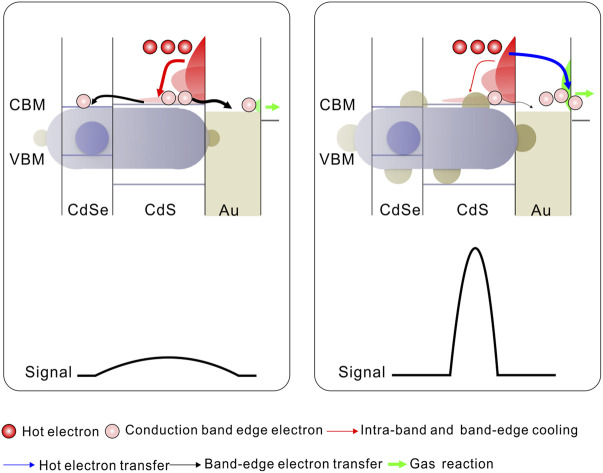

## Introduction

The efficient transduction of chemical information from volatile organic compounds (VOCs) into measurable signals under ambient conditions represents a grand challenge in materials science.^1–3^ Although chemoresistive sensors offer a promising platform for non-invasive diagnostics through breath analysis, owing to their potential for miniaturization and portability,^4–6^ they still face a certain number of challenges. Typically, thermal excitation sensors operate at high temperatures, introducing safety risks and power consumption issues and limiting their lifespan.^7^ Conversely, photoexcitation sensors can circumvent the issues associated with high-temperature operation.^8^ However, these sensors often struggle with the detection of trace gases, particularly weakly oxidizing and reducing long chain compounds at gas–solid interfaces, such as *trans*-2-nonenal–a C9 alkenal biomarker for non-small cell lung cancer–making ultra-trace detection at room temperature an enduring challenge for a long time.

A critical analysis of current photoactivated sensors reveals the core of the problem. While strategies like surface geometry optimization and localized surface plasmon resonance (LSPR) can enhance carrier generation,^9–12^ the subsequent charge transfer processes in conventional heterostructures occur on a nanosecond timescale.^11,13,14^ This timeframe is incontrovertible evidence that the transfer relies on the slow, diffusive migration of cooled band-edge electrons after they have fully relaxed to perform the sensing task. This paradigm inherently fails, as it reduces charge separation efficiency and cannot harness the initial, non-equilibrium energy of the photogenerated carriers in semiconductors, limiting the number and energy of electrons available for subsequent gas–solid interface reactions. Therefore, developing a new sensing architecture capable of intercepting and transferring these “hot” carriers before they cool has become a paramount objective.

**Scheme 1:**
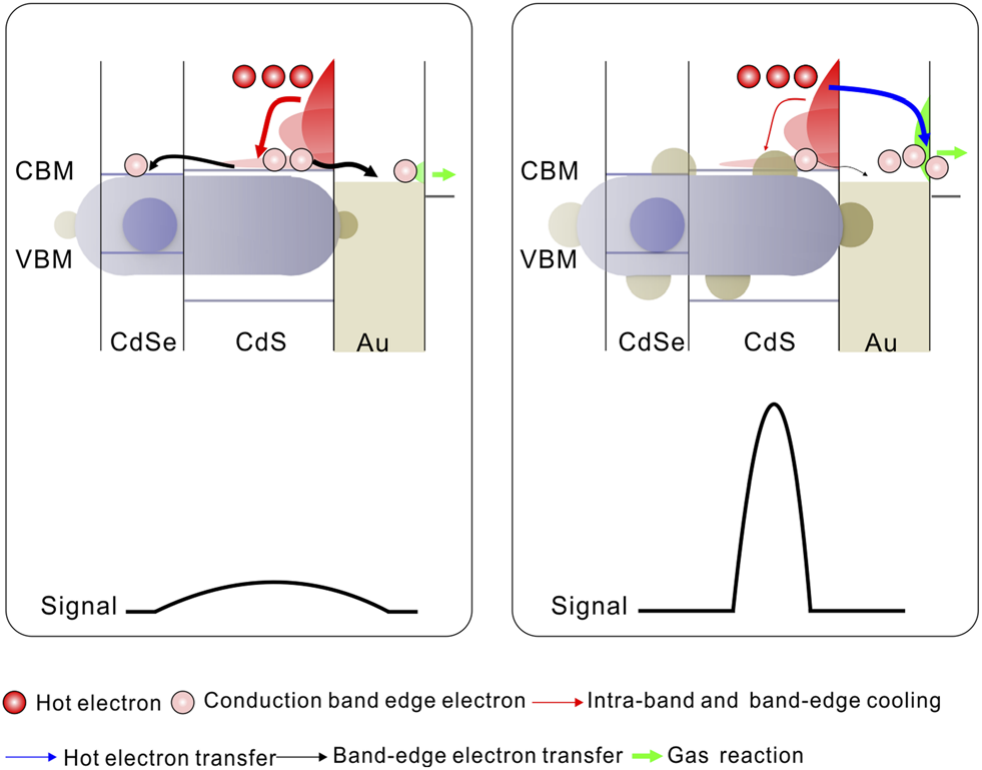
Schematic diagram of chemical physical processes for gas-sensing reactivity in CdSe@CdS-Au with two-tip Au domains and CdSe@CdS-Au with large multi-site Au domains and the corresponding detection signal variations. The term response signal refers to the resistance value.

Here, we introduce a new principle for gas sensing—non-equilibrium hot-electron-mediated chemoresistance—and embody it in a quantum-engineered CdSe@CdS-Au ternary heterostructure (Scheme 1). This platform is expressly designed to win the kinetic race against relaxation. The wide-gap CdS shell acts as a light-harvesting antenna, while the intrinsic quasi-Type-II band alignment with the CdSe core facilitates ultrafast, sub-picosecond exciton dissociation, pre-localizing the electron wavefunction near the heterostructure's surface.^15–17^ This step creates a high-flux source of electrons poised for extraction. Crucially, by strategically decorating the surface with Au nanodomains, we open a ballistic injection channel across the interfacial Schottky barrier. This architecture achieves an exceptionally rapid electron transfer with a 0.62 ps half-life, effectively capturing hot electrons mid-flight, before conventional cooling processes can occur. This direct harnessing of non-equilibrium carriers endows the sensor surface with more electrons available for subsequent gas–solid interface reactions and improved chemical activity. After functionalization with 4-bromobenzenethiol, the device achieves a record-breaking, sub-ppb limit of detection for *trans*-2-nonenal at room temperature. We term this new device class the “photoactivated hot electron chemoresistive sensor” to distinguish it from all conventional predecessors that rely on cooled carriers. To demonstrate its clinical potential, we integrated this material into a miniaturized six-channel sensor array, which successfully distinguishes multiple aldehyde compounds in simulated exhaled breath with remarkable specificity. This work establishes a new paradigm for chemical sensor design, offering a transformative approach that overcomes the fundamental limitations of cooled systems and opens new avenues for portable, high-precision disease screening.

## Results and discussion

### Synthesis and characterization of CdSe@CdS heterostructure rods integrated with Au nanoparticles

**Fig. 1:**
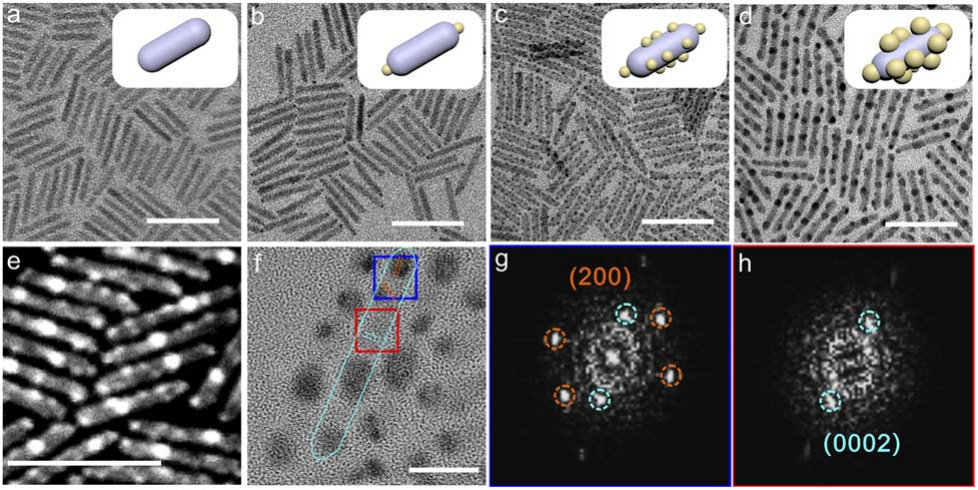
Morphological and structural characterization. (a) TEM images of CdSe@CdS. Scale bar, 50 nm. (b) TEM images of CdSe@CdS-TSAu. Scale bar, 50 nm. (c) TEM images of CdSe@CdS-MSAu. Scale bar, 50 nm. (d) TEM images of CdSe@CdS-MBAu. Scale bar, 50 nm. (e) HAADF image of CdSe @ CdS-MBAu. Scale bar, 50 nm. (f) High-resolution TEM image of CdSe@CdS-MBAu. Scale bar, 10 nm. (g) The FFT image corresponding to the blue region in (f). (h) The FFT image corresponding to the red region in (f).

CdSe/CdS heterostructure nanorods were synthesized using a seeded-type growth approach with minor modifications.^18,19^ By employing the thermal injection method, 2.4 ± 0.2 nm CdSe seeds were synthesized. The seeds exhibit a sharp exciton peak at 501 nm for 1S and an emission peak at 520 nm with a narrow full width at half maximum of 22 nm, respectively, indicative of homogeneous particle size distribution (Fig. S1). CdS grown at such a CdSe seed size exhibits a type-II band structure.^15–17^ Subsequently, high-quality CdSe/CdS nanorods were synthesized *via* high-temperature co-injection of a mixture of sulfur powder and the seeds. The resulting nanorods had lengths of 35 ± 3.1 nm and diameters of 4.6 ± 0.4 nm (Fig. 1a). The CdSe core was positioned between the 1/3 and 1/4 regions along the overall length of the CdS nanorods.^18^

Site-selective Au deposition on CdSe/CdS nanorods was achieved *via* the oleylamine reduction of gold chloride, following a modified procedure (see the SI for experimental details).^20,21^ By controlling the amount of precursor and reaction time, three distinct configurations of CdSe@CdS nanorods with Au domains were obtained: CdSe@CdS with two-tip small Au domains (CdSe@CdS-TSAu), CdSe@CdS with multi-site small Au domains (CdSe@CdS-MSAu), and CdSe@CdS with large multi-site Au domains (CdSe@CdS-MBAu). The sizes of the Au domains in the three samples are as follows: 1.7 ± 0.6 nm for CdSe@CdS-TSAu, 1.7 ± 0.6 nm for CdSe@CdS-MSAu, and 4.2 ± 0.5 nm for CdSe@CdS-MBAu (Fig. 1b–d and S2). In the XRD test, the successful growth of Au domains onto CdSe/CdS nanorods is revealed (Fig. S3). High annular dark-field scanning transmission electron microscopy (HAADF-STEM) revealed the contrasting Au domain growth on the CdSe/CdS nanorod body (Fig. 1e). High-resolution TEM (HR-TEM) confirmed the different lattice fringes from the *d* spacings of 2.4 Å for Au (200) planes and *d* spacings of 3.4 Å for CdS (0002) planes (Fig. 1f–h).^20,21^

### Charge carrier dynamics and electron transfer

**Fig. 2:**
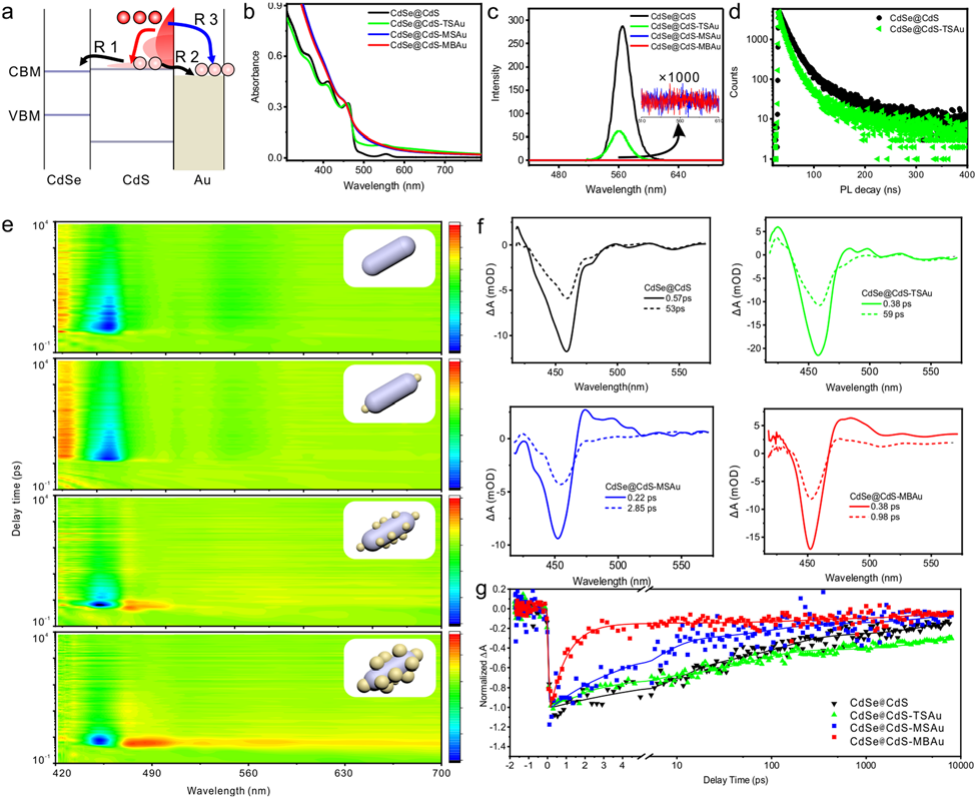
Steady-state spectrum and transient spectrum. (a) Schematic diagram of the physical process. (b) UV-vis absorption spectra of CdSe@CdS and heterostructures. (c) PL emission spectra of CdSe@CdS and heterostructures. The inset shows a localized zoom-in spectrum at a magnification of 1000. (d) Time-resolved fluorescence decay curves of CdSe@CdS and heterostructures. (e) Two-dimensional pseudo-color plots of the transient absorption spectra of CdSe@CdS and heterostructures. (f) Transient absorption spectra of CdSe@CdS and heterostructures. (g) Bleaching decay of CdSe@CdS and heterostructures at the CdS bandgap.

The photophysical processes under excitation are simplified as shown in Fig. 2a (for clarity, the hole trapping is not depicted). Fig. 2b implies that CdSe@CdS exhibits three distinct exciton absorption peaks at 470 nm, 415 nm, and 370 nm, respectively, which correspond to the 1Σ, 1Π, and higher excited state transitions 1Δ and 2Σ of the CdS nanorods.^22–24^ Additionally, a weak absorption peak at 550 nm is attributed to the 1*σ* transition of the CdSe core.^25^ Similarities exist between the absorption characteristics of the end-distributed system and those of CdSe@CdS, in which the primary difference lies in an overall increase in absorbance due to the absorption by Au particles.^26^ In the multi-site distribution system, the absorbance exhibits an increase and 1Π and 1*σ* transitions of CdS are obscured by the absorption of Au particles and the 1Σ transition of CdS presents a slight blue shift. This indicates that introducing gold domains, particularly in a multi-site arrangement, alters the excitonic transition characteristics of CdSe@CdS and, thereby, substantially enhances the plasmon-exciton coupling. Compared to CdSe@CdS, the end-distributed system exhibits partial quenching of both steady-state fluorescence and fluorescence lifetime (Fig. 1c and d). The quenching efficiency of steady-state fluorescence is higher than that of the fluorescence lifetime, indicating that the fluorescence quenching is attributed to a combination of electron transfer and static quenching.^27^ However, in the multi-site system, fluorescence is completely quenched, confirming that electrons in the CdS nanorods are completely transferred to the Au domains as opposed to residing in the CdSe core.

Charge carrier dynamics were monitored through broadband ultrafast TA spectroscopy with 400 nm excitation light from an 800 nm source. According to Fig. 2e–g, a prominent bleaching peak is observed at approximately 458 nm in the transient absorption spectra of CdSe@CdS and CdSe@CdS-TSAu. This can be attributed to exciton filling in the CdS shell. Observing the weaker bleaching signal at approximately 550 nm, the filling of exciton states in the CdSe core is reflected, primarily caused by the presence of conduction band electrons, resulting in negative absorption.^28^ Upon 400 nm excitation, selective excitation of CdS nanorod's light-harvesting antenna enhances exciton population in the CdSe core through energy transfer across the CdS shell.^29^ To examine electron transfer dynamics, we compared the decay kinetics of the approximately 458 nm bleaching signal in CdSe@CdS and CdSe@CdS-TSAu. The results indicate that no significant differences are observed over ∼8 ns even though the bleaching recovery of CdSe@CdS-TSAu is faster in ∼100 ps. This suggests that electron transfer from CdS to Au domains and from CdS to CdSe serves as a competing process affecting the overall electron transfer rate.

In the multi-site system, the samples exhibited a blue shift of the bleach signal, potentially due to the etching effect of Au growth on the CdS rods.^30^ No significant bleach signal from the CdSe core was detected since this feature was masked by the strong absorption of the Au domains. Analysis of the bleach dynamics revealed that the decay rates of CdSe@CdS-MSAu and CdSe@CdS-MBAu were notably faster than those of CdSe@CdS and CdSe@CdS-TSAu. By employing the half-life τ1/2 as the characteristic electron transfer time, we observed a significant reduction in the efficient electron transfer characteristic time within CdSe@CdS-MSAu and CdSe@CdS-MBAu to 5.5 ps and 0.62 ps (Table S1).^31^ Upon excitation of CdS at 400 nm, multiple physical processes can occur, including hot exciton (hot carrier) relaxation, Auger recombination, radiative recombination, and other non-radiative recombinations. Following the attachment of Au, an additional electron transfer pathway is incorporated. Compared to the control group that features gold at both ends, the electron transfer timescale of CdSe@CdS heterostructure rods integrated with multi-site gold was decreased by 9.6 times. To evaluate the rate of this electron transfer pathway, the rate in the presence and absence of gold was compared using a simplified two-channel model *K*_all_ = *K*_0_ + *K*_1_, where *K*_0_ represents the exciton lifetime without Au attachment, including electron transfer to CdSe, Auger recombination, radiative recombination, and other non-radiative recombination processes, and K_1_ serves as the electron transfer rate. The electron transfer rate from CdS to Au for CdSe@CdS-MSAu was obtained at 1.6 × 10^11^ s^−1^ (Table S2), which indicates the occurrence of partial transfer of hot electrons because the calculated electron transfer rate is higher than the Auger recombination rate (*K*_Auger_ ≈ 10^8^ − 10^10^ s^−1^).^31,32^ The electron transfer rate from CdS to Au for CdSe@CdS-MBAu was determined to be 1.60 × 10^12^ s^−1^ with an efficiency of 99%. This suggests the dominant role of hot electron transfer.^33–37^ This can be attributed to strong coupling between type-II band alignment of CdSe@CdS and multi-site distribution of large Au domains. The Type-II band alignment increases the lifetime of hot electrons through inducing spatial separation of electrons and holes. The multi-site distribution of Au domains shortens the electron transfer pathway, while the nanometer-scale control of Au domains results in a great transfer cross-section and high potential. This, in turn, facilitates hot electron transfer through ballistic transport, tunneling effects or energy coupling, bypassing the need for in-band electron cooling prior to transfer.

### The detection of non-small cell lung cancer biomarkers

**Fig. 3:**
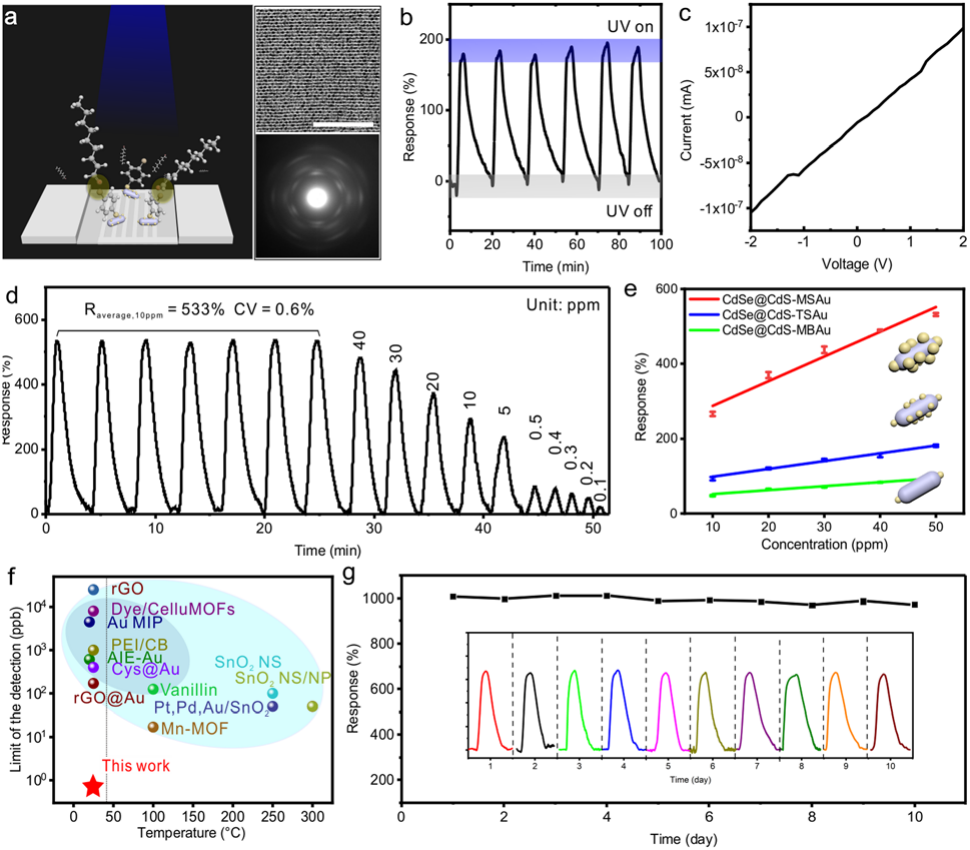
Detection performance of *trans*-2-nonenal. (a) Schematic diagram of the sensor with the modified sensing materials with 4-bromobenzenethiol and interdigital electrodes for *trans*-2-nonenal. A typical TEM image of the CdSe@CdS-MBAu assembly at the top right and small angle electron diffraction at the bottom right. Scale bar, 100 nm. (b) Response curve of the sensor under UV irradiation. (c) Volt-ampere characteristic curve. (d) Concentration-dependent response-recovery curves. (e) Response-concentration plots of CdSe@CdS, CdSe@CdS-TSAu, CdSe@CdS-MSAu and CdSe@CdS-MBAu sensors. (f) Comparison of limits of detection for *trans*-2-nonenal, homologous compounds and C9 alkanals in optical sensors (dark blue shadow) and chemoresistive sensors (light blue shadow). According to the IEC 60601-1-11 standard for medical electrical equipment in home healthcare environments, accessible surface temperature must not exceed 43 °C (left side of the dotted line). (g) Cyclic response curves of sensors. The term response signal refers to the resistance value.

To facilitate the detection of non-small cell lung cancer biomarker *trans*-2-nonenal by employing a photoactivated chemoresistive sensor, we modified the sensitive materials with 4-bromobenzenethiol, a chemical selector known to form strong halogen interactions with electronegative elements, including carbonyl oxygen (Fig. 3a).^31,38^ The successful modification was confirmed by the characteristic stretching vibration peak of observed 4-bromobenzenethiol (Fig. S4). The modified sensitive material was assembled into a thin film and transferred onto interdigitated electrodes for gas response testing (Fig. S5). When irradiated with 1 W of 365 nm light, the modified sensor exhibited a notable photoelectric response and demonstrated a linear voltage–current relationship in the range of 0 to 2 V, which indicated the effective operation of the developed photoactivated chemoresistive sensor for subsequent gas response testing (Fig. 3b and c).

The CdSe@CdS-MBAu photoactivated chemoresistive sensor exhibited sensitive and reversible responses to *trans*-2-nonenal in a concentration range from 10 to 50 ppm (Fig. 3d). CdSe@CdS-MBAu demonstrated the highest gas response among the tested sensitive materials at a concentration of 1 ppm and achieved a detection limit of 0.70 ppb (Fig. 3e, S6–S9 and Table S3), which is the lowest reported among different materials that detect *trans*-2-nonenal, homologous compounds and C9 alkanals (Fig. 3f and Table S4). This enhanced performance is attributed to the occurrence of hot electron transfer, allowing more electrons to engage in subsequent surface reactions or conductivity processes. By capturing photo-generated electrons, oxygen molecules adsorbed on the surface of gold domains or CdS can form superoxide anions (O_2_^−^). When *trans*-2-nonenal interacts with such oxygen species, the species desorb from the sensor surface and then release electrons back into the material, thereby resulting in changes in conductivity. The CdSe@CdS-MBAu sensor demonstrated good selectivity for *trans*-2-nonenal among the common gases found in exhaled breath (Fig. S10). However, an increase in humidity raised the detection limit, indicating that dehydrating the target gases plays a necessary role in maintaining the optimal sensor performance (Fig. S11). Given the long-term stability of the CdSe@CdS-MBAu sensor, consistent response amplitude over a 10-day observation period was confirmed, which indicated stable performance (Fig. 3g).

### Portable photoactivation chemoresistive chips for the identification of specific non-small cell lung cancer biomarkers

**Fig. 4:**
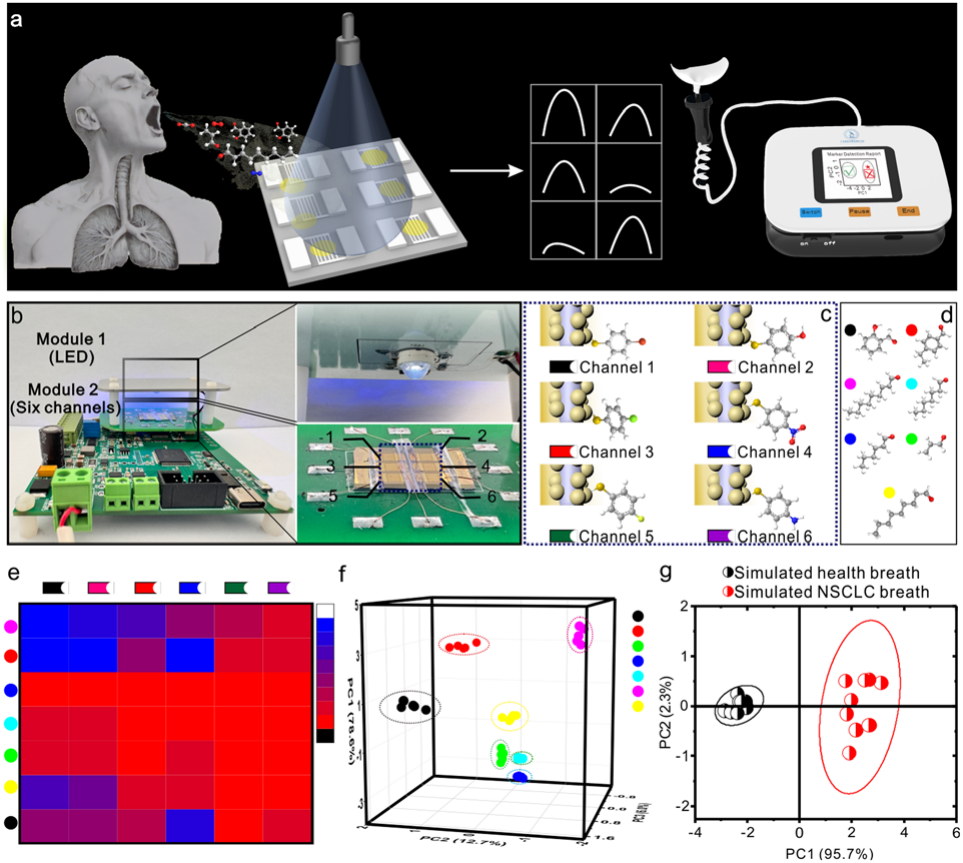
A six-channel UV-activated sensor chip for a portable device for identifying non-small cell lung cancer biomarkers. (a) Illustration of screening of non-small cell lung cancer. (b) Photos of the designed circuit board with six-channel data collection support module. (c) CdSe@CdS-MBAu with a 4-bromobenzenethiol ligand, CdSe@CdS-MBAu with a 4-chlorobenzenethiol ligand, CdSe@CdS-MBAu with a 4-fluorobenzenethiol ligand, CdSe@CdS-MBAu with a 4-hydroxybenzenethiol ligand, CdSe@CdS-MBAu with a 4-nitrobenzenethiol ligand, and CdSe@CdS-MBAu with a 4-aminobenzenethiol ligand as gas-sensitive materials for six channels. (d) Volatile aldehyde compounds in exhaled breath including *trans*-2-nonenal, *trans*-2-hexenal, heptanal, nonanal, acrolein, *p*-ethylbenzaldehyde and salicyladehyde. (e) Heat map of the sensor-specific response for volatile aldehyde compounds in exhaled breath. (f) PCA plot of sensor response to *trans*-2-nonenal analogs. (g) PCA of a set of simulated healthy breath samples and non-small cell lung cancer breath samples.

To satisfy the demand for portable devices in point-of-care testing, we developed a compact circuit board and illumination module for the photoactivation chemoresistive sensor (Fig. 4a, b and S12). Due to the sensor's ultra-low detection limit, the potential identification of *trans*-2-nonenal, a biomarker for non-small cell lung cancer, in exhaled breath is enabled. However, minimizing false-positive results due to cross-reactivity with *trans*-2-nonenal analogs remains essential in broadening its practical applications. Accordingly, CdSe@CdS-MBAu was modified with selected ligands, including 4-bromobenzenethiol, 4-chlorobenzenethiol, 4-fluorobenzenethiol, 4-hydroxybenzenethiol, 4-nitrobenzenethiol, and 4-aminobenzenethiol, which created a six-channel sensor array to enable cross-reactive responses for discriminating volatile aldehyde compounds in exhaled breath (Fig. 4b–d).

Fig. 4e exhibits the raw sensor responses and corresponding heatmap visualization (Fig. S13). As observed in the results, the six-channel VOC sensor array generates a unique response pattern for each analyte in just 1 min of exposure. To streamline data interpretation, principal component analysis (PCA) was employed for reducing dimensionality. With the first three principal components capturing more than 95% of the total variance, we accurately distinguished all seven plant VOCs at 1 ppm from the control (N_2_) out of 35 trials (Fig. 4f and S14). An overall classification accuracy of 97.1% (34/35) was then yielded, which demonstrated that six-channel sensor design is well-suited for practical detection requirements. Besides, a simulated exhaled breath mixture, comprising nitrogen (75%), oxygen (16%), carbon dioxide (4%), water vapor (4.06%), and noble gases (0.94%), was prepared with *trans*-2-nonenal at 1.4 ppb concentrations. To ensure optimal testing conditions, water vapor was removed from the simulated gas through the utilization of a calcium chloride desiccant. The first two PCA components accounted for 97.9% of the variance, which effectively distinguished between simulated lung cancer and healthy profiles with distinct and non-overlapping clusters (Fig. 4g and S15).

## Conclusions

In conclusion, this work establishes and experimentally validates the principle for chemical sensing: non-equilibrium hot-electron-mediated chemoresistance and photoactivation hot electron chemoresistive sensors were achieved. By rationally designing a quantum-engineered CdSe@CdS-Au heterostructure, we successfully circumvent the fundamental performance limitations imposed by carrier thermalization. By precisely controlling the distribution and size of the Au domains, the electron transfer half-life was reduced to 0.62 ps, facilitating efficient hot electron transfer and enhancing the participation of electrons in gas–solid interface reactions. Our sensor achieves the record-breaking detection of the non-small cell lung cancer biomarker, *trans*-2-nonenal, at a limit of 0.70 ppb. Furthermore, a portable six-channel sensor array achieved 97.9% accuracy in distinguishing simulated healthy and cancerous breath profiles, showcasing a significant leap towards clinical applications. This work establishes a transformative design paradigm that shifts the focus from managing cooled carriers to directly utilizing energetic, non-equilibrium hot electrons, opening new avenues for developing the next generation of ultra-sensitive portable devices for non-invasive medical diagnostics, environmental monitoring, and beyond.

## Author contributions

T. W., X. L. and S. Y. conceived the idea for this project. X. L. and J. L. synthesized and characterized CdSe@CdS. X. L., J. L., R. L., S. J., C. L., D. B., S. Z., R. L. and P. W. carried out the characterization analyses. W. Z. and S. Y. performed the steady-state spectrum and transient spectrum. X. L. J. L., R. L., L. H., M. Y., X. L. and Z. X. carried out detection of *trans*-2-nonenal. X. L., W. Z., L. R., S. Z., S. Y. and T. W. wrote drafts of the paper. X. L., S. Y. and T. W. directed the project. All the authors participated in evaluating the results and commented on the manuscript.

## Conflicts of interest

There are no conflicts to declare.

## Supplementary Material

SC-OLF-D5SC05009J-s001

## Data Availability

All the data have been presented in the manuscript and SI. Materials and characterizations, experimental section, structural and morphological characterization, parametric fitting of bleaching kinetics to transient absorption spectra, simplified two-channel model the rate and efficiency of electron transfer, schematic diagram of interface assembly for the sensor, sensor response to trans-2-nonenal, detection limits of various sensors, selectivity of the sensor, effect of humidity on sensor, schematic diagram of the printed circuit board module for the sensor and scree plot of PCA. See DOI: https://doi.org/10.1039/d5sc05009j.
