# Supplementary material for: Harnessing non-equilibrium hot electrons in a quantum-engineered ternary heterostructure for sub-ppb C9 biomarker detection
Source: Chem Sci. 2025 Aug 18;16(37):17200–6. doi: 10.1039/d5sc05009j (PMC12394916; doi:10.1039/d5sc05009j)
Supplement: SC-016-D5SC05009J-s001 [file SC-016-D5SC05009J-s001.pdf]

Supplementary Information (SI) for Chemical Science.
This journal is © The Royal Society of Chemistry 2025

Supplementary Information

Harnessing Non-Equilibrium Hot Electrons in a Quantum-Engineered Ternary Heterostructure for Sub-ppb C9 Biomarker Detection

Table of Contents

Section S1. Materials and characterizations 3
S1.1 Materials 3
S1.2 Characterizations 3
S1.3 Femtosecond time-resolved transient absorption spectroscopy 3
Section S2. Experimental section 4
S2.1 Synthesis of CdSe 4
S2.2 Synthesis of CdSe@CdS nanorods 4
S2.3 Au depositions on CdSe@CdS nanorods 4
S2.4 Modification of CdSe@CdS-MBAu materials 5
S2.5 Preparation of gas-sensitive chips 5
S2.6 Simulated gas composition 5
Section S3. Structural and morphological characterization 6
Section S4. Parametric fitting of bleaching kinetics to transient absorption spectra 8
Section S5. Simplified two-channel model the rate and efficiency of electron transfer 9
Section S6. Schematic diagram of interface assembly for the sensor 10
Section S7. Sensor response to trans-2-nonenal 11
Section S8. Detection limits of various sensors 13
Section S9. Selectivity of the sensor 15
Section S10. Effect of humidity on sensor 16
Section S11. Schematic diagram of the printed circuit board module for the sensor 17
Section S12. Scree plot of PCA 18
References 20

Section S1. Materials and characterizations

S1.1 Materials

Trioctyl phosphorus ($C_{24}H_{51}OP$, 99.9%), cadmium oxide (CdO, 99.99%), selenium powder (Se, 99.99%), sulfur (S, 99.99%), dodecyltrimethylammonium bromide ($C_{15}H_{34}BrN$, 99.99%), gold chloride ($AuCl_3$, 99%), and 4-bromothiophenol (C_6H_5BrS, AR) were procured from Heowns. Octadecylphosphonic acid ($C_{18}H_{39}O_3P$, 98%) was obtained alongside highly pure compounds such as 4-fluorothiophenol (C_6H_5FS, 98%), 4-hydroxythiophenol (C_6H_6OS, 98%), 4-aminothiophenol (C_6H_7NS, 98%). Additionally, heptanal ($C_7H_{14}O$, 98%) and nonenal ($C_9H_{16}O$, AR) were acquired from Alfa. Acrolein (C_3H_4O, 99.95%) was sourced from Aladdin. Furthermore, trioctylphosphine ($C_{24}H_{51}P$, 90%), toluene (C_7H_8, 95%), hypophosphorous acid (H_3PO_2, 55%), oleylamine ($C_{18}H_{37}N$, 85%), ethanol (C_2H_6O, 99.95%), ethyl acetate ($C_4H_8O_2$, 98%), and hexenal ($C_6H_{10}O$, 50%) were purchased from Aladdin. 4-Chlorothiophenol (C_6H_5ClS, 98%) was purchased from Acros. Moreover, the following notable acquisitions include: 4-nitrobenzenethiol ($C_6H_5NO_2S$, 99%), trans-2- nonanal ($C_6H_{18}O$, 95%), salicylaldehyde ($C_7H_6O_2$, 99.5%) were purchased from Innochem. Finally, 4-ethylbenzaldehyde ($C_9H_{10}O$, 95%) was purchased from Tci.

S1.2 Characterizations

The crystal structure of the materials was characterized by a smart powder x-ray diffractometer (Ultimal V, Rigaku corporation, Japan). The microstructural morphology was examined with a transmission electron microscope (TECNAI G2 Spirit TWIN, FEI, USA) equipped with a LaB6 filament and a high-resolution transmission electron microscope (Talos F200 X, FEI, USA). Steady-state absorption spectra were measured utilizing a UV-VIS/NIR spectrophotometer (Lambda750 UV/VIS/NIR, Perkin Elmer Perkin Elmer, USA). The detection and quantification of specific gases in the environment were performed using an Intelligent Gas Analyzer System (CGS-8, Beijing Zhongju Zhongke Technology Co. Ltd., China). Infrared testing was conducted employing a Fourier Transform Infrared Spectrometer (IRTracer-100, Shimadzu Corporation, Japan), accompanied by an infrared microscope model AIM-9000. Fluorescence spectroscopy detection was carried out using a spectrofluorometer (RF-6000, Shimadzu Corporation, Japan), featuring a laser source operating at 532 nm wavelength. Additionally, fluorescence lifetime measurements were executed with a steady-state transient fluorescence spectrometer (FLS-980, Edinburgh Instruments, UK), utilizing an excitation wavelength of 360 nm.

S1.3 Femtosecond time-resolved transient absorption spectroscopy

The experimental setup of the pump−probe femtosecond transient absorption spectroscopy (TAS) was reported in previous work. [1] In summary, 800 nm fundament pulses were generated using the Spitfire pro system (Spectra Physics) with a pulse duration of 50 fs width duration (full width at half-maximum, FWHM). The white-light continuum probe pulses (350−700 nm) were generated by focusing 800 nm pulses into a 2.0 mm thick CaF_2 window. The 800 nm pulses to generate white-light probe pulses were delayed with respect to the pump pulses using a translation stage (Newport) before generating white light. To eliminate any potential polarization-dependent effects, the pump pulses were linearly polarized at the magic angle concerning the probe pulses. The typical instrument response function (IRF) was estimated to be 90 fs (FWHM), based on measurements of the Kerr effect in n-hexane. All samples were analyzed at room temperature, with a pump power of 270 μJ/cm² at a wavelength of 400 nm. Transient absorption signals were fitted utilizing both global and target method. [2, 3]

Section S2. Experimental section

S2.1 Synthesis of CdSe

CdSe seeds were synthesized following a method described in the literature with minor modification.[4, 5] Briefly, 3.0 g of trioctylphosphine oxide (TOPO), 0.28 g of n-octadecyl phosphoric acid (ODPA), and 0.06 g of cadmium oxide (CdO) were rapidly added to a 50 ml three-necked flask under magnetic stirring and reflux conditions. The solution was subjected to vacuum evacuation at room temperature for 5 minutes, after which it was heated to 150°C and maintained under vacuum for an additional hour.

Subsequently, 0.058 g of Se (selenium powder) was combined with 0.5 ml of TOPO and thoroughly mixed until dissolved by sonication, resulting in a completely clarified solution. Once TOPO transitioned to a liquid state within the three-necked flask, nitrogen gas was introduced into the system while raising the temperature to 300 °C. After achieving complete clarification of the solution, 1.8 ml of TOPO solution was injected into the three-necked flask using a syringe; thereafter, the temperature was adjusted to reach 380 °C. Upon reaching this target temperature, the previously prepared Se-TOPO mixture was swiftly injected via syringe into the reaction vessel, causing an immediate color change in the solution to green. The resultant mixture was then rapidly cooled using acetone until it reached room temperature; subsequently, 4 ml of toluene were added before precipitating with ethanol. The synthesized CdSe quantum dots were isolated by centrifugation at 6000 rpm for five minutes, which allowed for the collection of the supernatant fluid. Ethanol was then introduced into this supernatant to ensure complete precipitation; any remaining precipitate underwent further centrifugation at 7000 rpm for five minutes for collection purposes. Finally, the collected precipitate was dispersed in 1 ml of TOPO solution before being sealed and placed in a vacuum drying oven for storage.

S2.2 Synthesis of CdSe@CdS nanorods

CdSe@CdS nanorods were synthesized using a previously published method with modification. [4, 5] To initiate the synthesis, 0.075 g of CdO, 0.29 g of ODPA, 0.08 g of hypophosphorous acid (HPA), and 3 g of TOPO were meticulously combined in a 50 ml three-necked flask to achieve a homogeneous mixture. This was subjected to vacuum pumping for a duration of 10 minutes at ambient temperature, subsequently elevating the temperature to 150°C while continuing the vacuum process for an additional hour. The synthesis proceeded within the confines of this three-necked flask. In a separate operation, 0.12 g of high-purity sulfur was introduced into 1.8 ml of an oxygenated solution contained in a distinct 10 ml three-necked flask under magnetic stirring at room temperature. The solution underwent vacuum treatment for half an hour before being gently heated to 60 °C until complete dissolution of the high-purity sulfur powder was achieved; thereafter, an injection of 0.1 ml CdSe seeds ensued, allowing for a reaction period lasting ten minutes followed by nitrogen gas purging as precautionary back up. Once thoroughly dispersed within another designated 50 ml three-necked flask, nitrogen gas was continuously passed through it while raising the temperature incrementally to reach a state where the solution became entirely colorless. Subsequently, the temperature was further elevated to 380°C while simultaneously injecting an additional 1.8 ml of TOPO into the flask via syringe, which caused a cooling effect on the surrounding temperatures. "Following this adjustment phase, during which the temperature stabilized to approximately 350 °C, 10 ml of previously prepared CdSe quantum dot solution was rapidly injected into the vessel using a glass syringe. Maintaining these conditions for five minutes allowed optimal interaction, after which the solution was quickly cooled using an acetone solution. Upon reaching room temperature, 4 mL of toluene was added to the system. Ethanol solution was then used to facilitate the complete precipitation of the synthesized CdSe@CdS nanorods. The collection process was carried out through centrifugation at 7000 rpm for five minutes, yielding concentrated precipitates. These precipitates were then re-dispersed in 2 mL of pure toluene solvent. Finally calibrated and diluted accordingly led us towards achieving desired concentrations set forth at precisely 10 mg/ml concentration levels established throughout our resultant CdSe@CdS nanorod solutions produced during these intricate synthetic procedures undertaken herein described above.

S2.3 Au depositions on CdSe@CdS nanorods

A 10 ul of trioctylamine ligand was added to 500 ul of the previously prepared CdSe@CdS nanorods and allowed to grow for 20 minutes. Subsequently, anhydrous ethanol was introduced to render the solution completely turbid. The supernatant was then removed by centrifugation at 7,000 rpm for 5 minutes, and toluene was added to re-disperse the solution. This process was repeated in order to obtain CdSe@CdS nanorods coated with trioctylamine ligand. To a mixture containing 3.73 mL of toluene, varying masses of $AuCl_3$ (8mg, 12 mg, and 16 mg) along with 88 mg of DTAB were added. Following this addition, 260 uL of oleylamine ligand was incorporated into the mixture. The resulting solution was sonicated for

approximately 15 minutes until fully dissolved. Finally, the solution underwent filtration through a PTFE membrane with a diameter of 0.2 μm in order to yield a clear light gold-colored solution. The CdSe@CdS nanorod solution was heated to 60 °C using a water bath. The gold precursor solution was rapidly injected into this heated environment via syringe, and the reaction proceeded under controlled conditions for various durations (1 hour, 1.5 hours, and 2 hours) at maintained temperature before being transferred into a centrifuge tube. Ethyl acetate was subsequently added to the centrifuge tube followed by centrifugation at a speed of 9,000 revolutions per minute for ten minutes in order to precipitate solids. Those solids were then redispersed in toluene.

S2.4 Modification of CdSe@CdS-MBAu materials

The preparation involved mixing 125 ul of CdSe@CdS nanorods, which had gold domains attached to the rods, with 30 mg of 4-bromothiophenol dissolved in 500 ul of toluene solution. The resulting mixture was subjected to ultrasonication for 20 minutes and subsequently allowed to stand for 8 hours. This process yielded CdSe@CdS-MBAu modified with 4-bromothiophenol. Following a similar procedure, modifications of CdSe@CdS-MBAu were carried out using 4-chlorobenzenethiol, 4-fluorobenzenethiol, 4-hydroxybenzenethiol, 4-nitrobenzenethiol, and 4-aminobenzenethiol respectively.

S2.5 Preparation of gas-sensitive chips

The prepared CdSe@CdS-MBAu modified with 4-bromothiophenol, with gold domains attached to the reagents, were subjected to centrifugation at 1400 revolutions per minute for a duration of 18 minutes. The supernatant was subsequently removed. The precipitate was then dissolved in 20 ul of toluene, and a volume of 2 ul from this solution was carefully deposited onto a purchased forked finger electrode. This assembly was dried using a wash ball to yield the gas-sensitive gas-sensitive chips. Additionally, modified CdSe@CdS-MBAu were synthesized using other ligands such as 4-chlorothiophenol, 4-fluorothiophenol, 4-hydroxythiophenol, 4-nitrothiophenol, and 4-aminothiophenol, following the same procedure outlined above for gas-sensitive chips.

S2.6 Simulated gas composition

A simulated exhaled gas mixture was prepared, comprising nitrogen (75%), oxygen (16%), carbon dioxide (4%), water vapor (4.06%), and trace gases (0.94%). The flow rates of each component were meticulously calibrated to achieve the desired proportions. For instance, a flow meter was employed to adjust the flow rates of nitrogen, oxygen, carbon dioxide, and water vapor to ensure their respective concentrations reached 75%, 16%, 4%, and 4.06%. The concentration of trace gases was fine-tuned using precise gas flow controllers to maintain a level of 0.94%. To guarantee uniform mixing, the gases were combined utilizing a gas mixer. An appropriate mixing duration could be established within the mixer to enhance homogeneity among the components. Water vapor was introduced into the gas mixture via an evaporator or water bath in order to attain the specified concentration of 4.06%. Throughout this process, it was ensured that both temperature and humidity levels of the mixed gas remained within suitable ranges during the introduction of water vapor. Finally, the resultant mixed gas was collected into specialized gas sampling bags for further analysis or use.

Section S3. Structural and morphological characterization

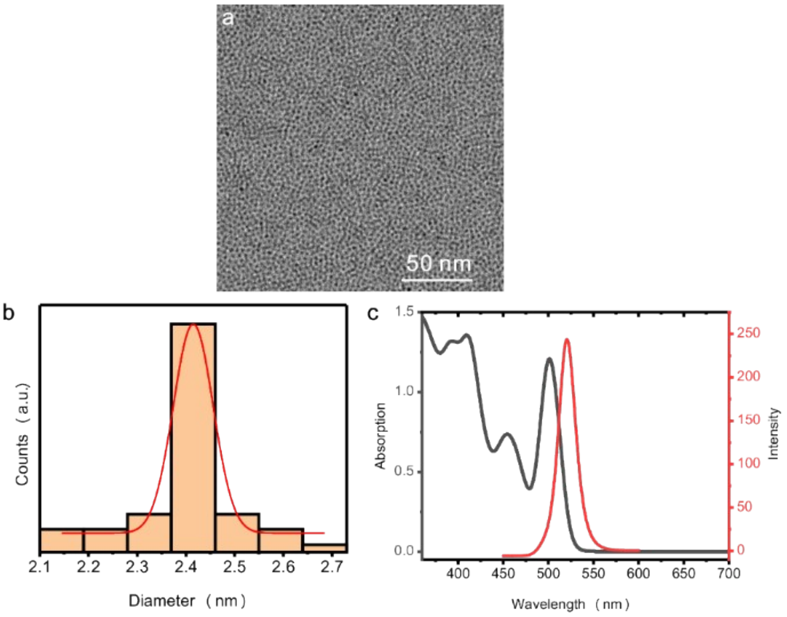

Figure. S1 Morphological and structural characterization of CdSe. (a) TEM of CdSe quantum dots, scale bar is 50 nm. (b) The statistical distribution of diameter. (c) Steady-state absorption spectroscopy (black) and steady-state fluorescence spectroscopy (red).

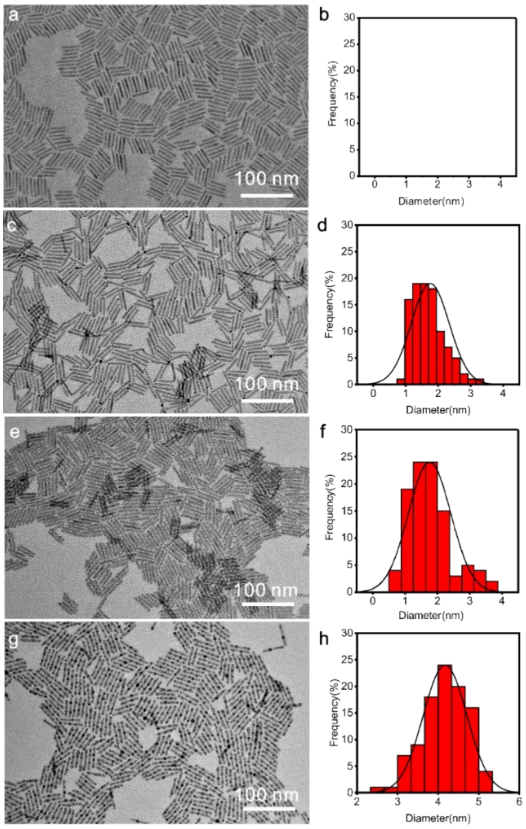

Figure S2 (a) TEM of CdSe@CdS, scale bar is 100 nm. (b) The statistical distribution of diameter of Au in CdSe@CdS. (c) TEM of CdSe@CdS -TSAu, scale bar is 100 nm. (d) The statistical distribution of diameter of Au in CdSe@CdS-TSAu. (e) TEM of CdSe@CdS-MSAu, scale bar is 100 nm. (f) The statistical distribution of diameter of Au in CdSe@CdS-MSAu. (g) TEM of CdSe@CdS-MBAu, scale bar is 100 nm. (h) The statistical distribution of diameter of Au in CdSe@CdS-MBAu.

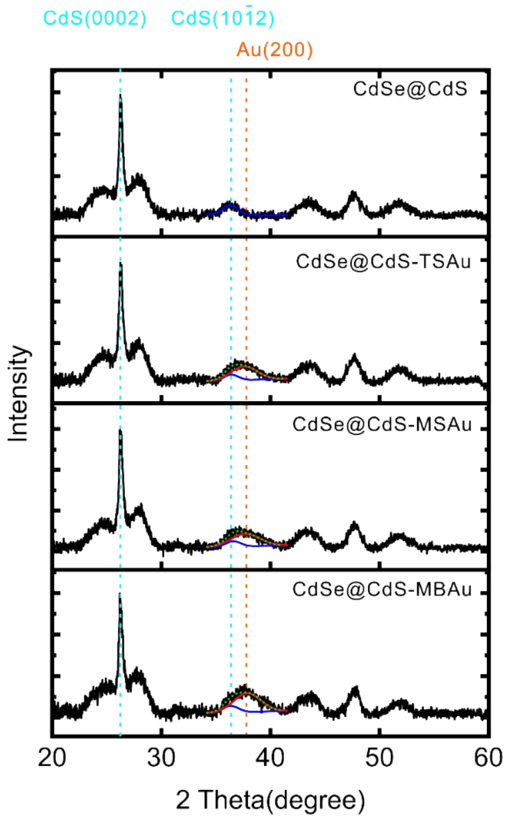

Figure S3. XRD analysis of CdSe@CdS, CdSe@CdS-TSAu, CdSe@CdS-MSAu, and CdSe@CdS-MBAu.

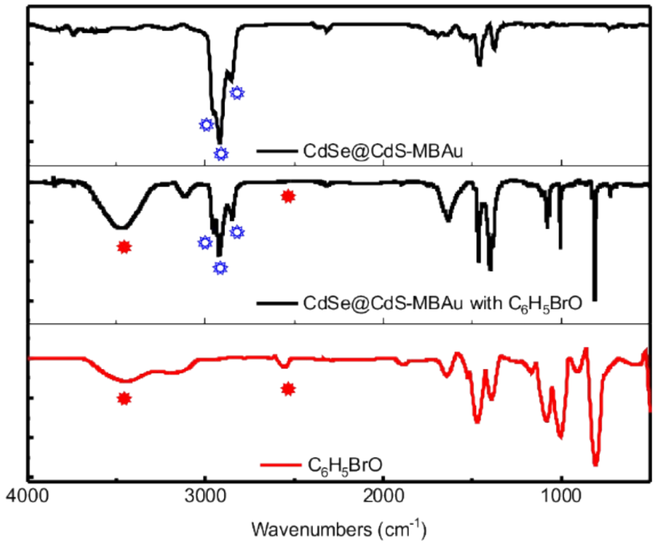

Figure S4. FT-IR spectra of CdSe@CdS-MBAu, CdSe@CdS-MBAu with C_6H_5BrO, and C_6H_5BrO.

Section S4. Parametric fitting of bleaching kinetics to transient absorption spectra

Table S1. Fitting parameters of sample bleaching attenuation kinetics

Sample	τ_1(ps) A1(%)	τ_2(ps) A2(%)	τ_3(ns) A3(%)	$\tau_{1/2}$(ps)
CdSe@CdS	6.8±1.9	107.9±30.3	>4	40
	40	32	28	
CdSe@CdS-TSAu	1.4±0.5	49.7±8.6	>4	53
	30	30	40	
CdSe@CdS-MSAu	3.9±1.0	94.3±58.2	>4	5.5
	65	20	15	
CdSe@CdS-MBAu	0.74±0.15	92.7±84.9	>4	0.62
	88	4	8	

Note

The data in Table S1 is obtained by fitting the decay curves using a 3-exponential method.

$$\Delta OD(t) = \sum_{i=1}^{3} A_i e^{\frac{-t}{\tau_i}}$$

Section S5. Simplified two-channel model the rate and efficiency of electron transfer

Table S2. Simplified two-channel model for calculating the rate and efficiency of electron transfer from CdS to Au.

Sample	$K_{all}(s^{-1})$	$K_0(s^{-1})$	$K_1(s^{-1})$	$P_1(\%)$
CdSe@CdS	2.50×10^{10}	2.50×10^{10}	0	-
CdSe@CdS-TSAu	1.89×10^{10}	2.50×10^{10}	-0.61×10^{10}	-
CdSe@CdS-MSAu	1.82×10^{11}	2.50×10^{10}	1.57×10^{11}	86.26
CdSe@CdS-MBAu	1.62×10^{12}	2.50×10^{10}	1.60×10^{12}	98.76

Section S6. Schematic diagram of interface assembly for the sensor

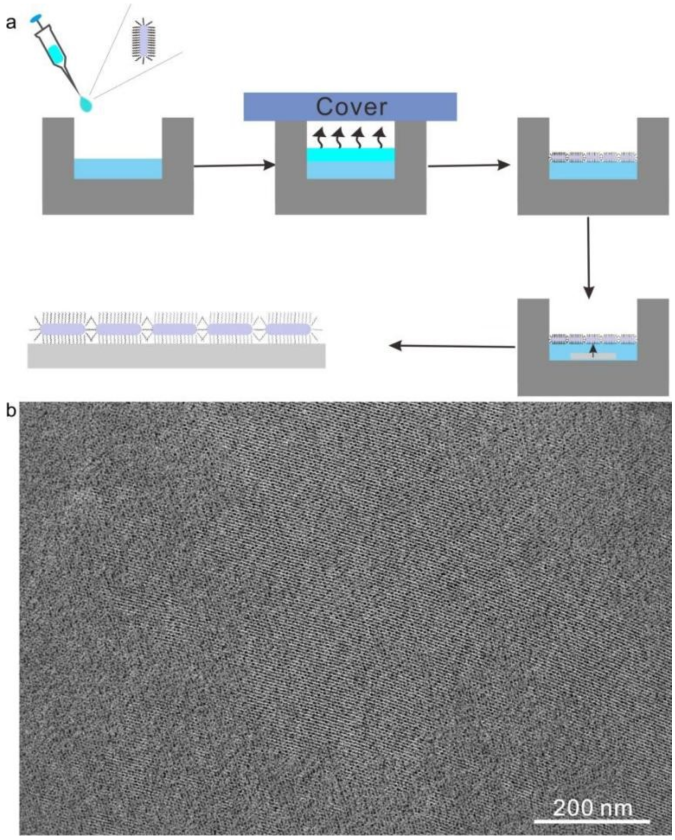

Figure S5. (a) Schematic diagram of the interface assembly with CdSe@CdS-MBAu nanostructures of 4-bromobenzenethiol molecules. (b) TEM of CdSe@CdS-MBAu with 4-bromobenzenethiol, scale bar is 200 nm.

Section S7. Sensor response to trans-2-nonenal

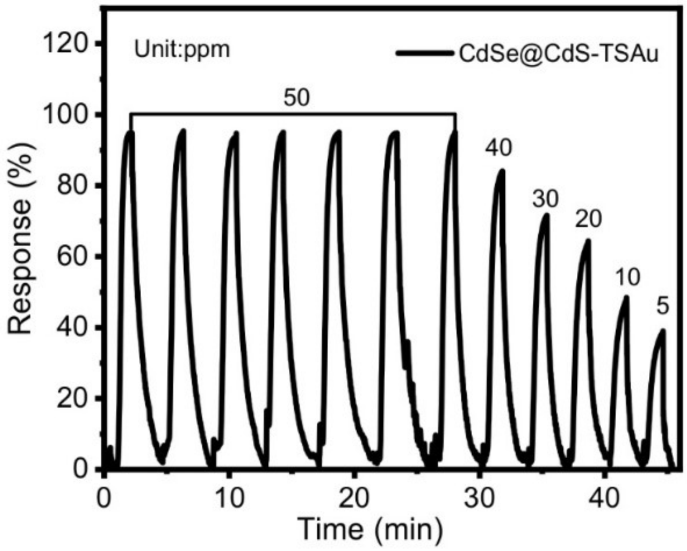

Figure S6. Graph of response curves of CdSe@CdS-TAu sensors to trans-2-nonenal at different concentrations of 50 ppm, 40 ppm, 30 ppm, 20 ppm, 10 ppm, and 5 ppm. The term response signal refers to the resistance value.

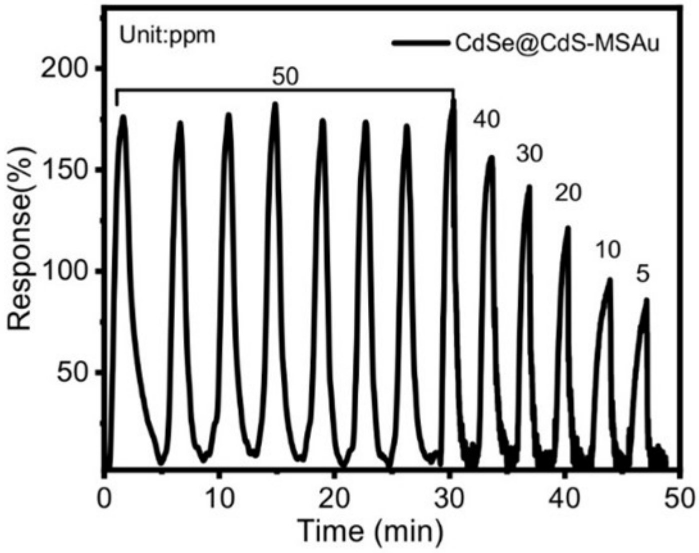

Figure S7. Response curves of CdSe@CdS-MSAu to trans-2-nonenal at concentrations of 50 ppm, 40 ppm, 30 ppm, 20 ppm, 10 ppm, and 5 ppm. The term response signal refers to the resistance value.

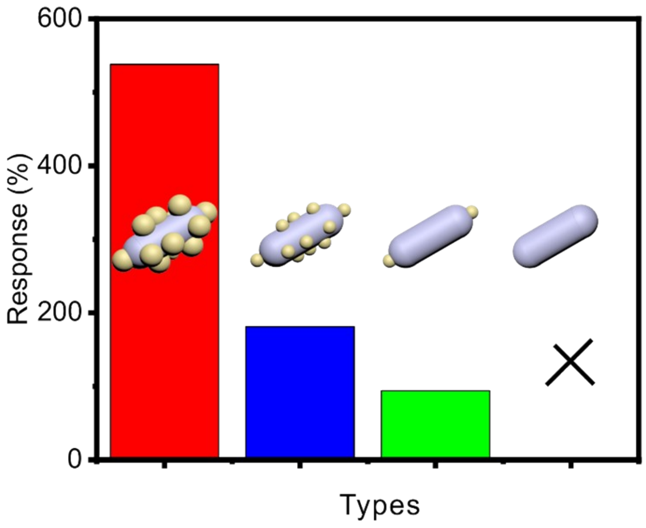

Figure S8. Comparison of response at 50 ppm of CdSe@CdS, CdSe@CdS-TSAu, CdSe@CdS-MSAu and CdSe@CdS-MBAu sensors. The term response signal refers to the resistance value.

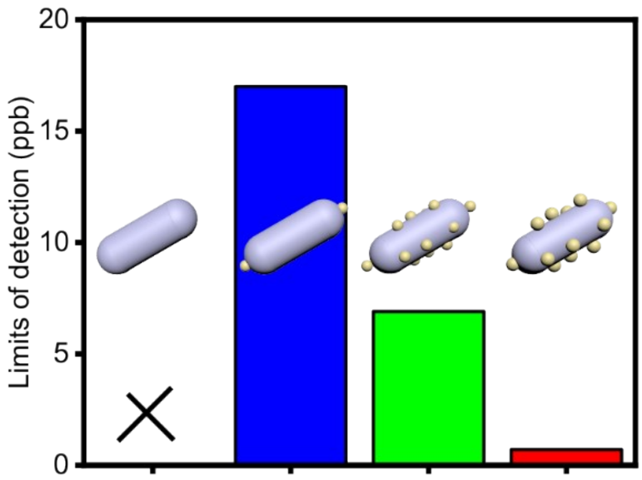

Figure S9. Limit of the detection of CdSe@CdS, CdSe@CdS-TAu, CdSe@CdS-MSAu, CdSe@CdS-MBAu for trans-2-nonenal.

Table S3. Limits of detection (LODs) of trans-2-nonenal for three CdSe@CdS-Au sensors.

Sample	R^2	Slope	Intercept	LOD（ppb）
CdSe@CdS-TSAu	0.97	1.06	41.33	49.00
CdSe@CdS-MSAu	0.97	2.08	77.73	6.90
CdSe@CdS-MBAu	0.99	6.62	220.73	0.70

Section S8. Detection limits of various sensors

Table S4. Comparison of limits of detection for trans-2-nonenal, homologous compounds and C9 alkanals in optical sensors and chemoresistive sensors.

Gas type	Sensor type	Temperature (℃)	Limit of detection	Reference	Date
Nonanal	Chemical resistance	250	50 ppb	DOI:10.5162/imcs2012/6.3.4	2012[6]
Trans-2-hexenal	Fluorescence	20	610 ppb	DOI:10.1016/j.jcis.2024.05.233	2014[7]
Nonanal	Chemical resistance	300	50 ppb	DOI:10.1038/srep10122.	2015[8]
Nonanal	Chemical resistance	250	100 ppb	DOI:10.1111/ijac.13154	2018[9]
Trans-2-hexenal	Colorimetric	25	400 ppb	DOI:10.1038/s41477-019-0476-y	2019[10]
Nonanal	Chemical resistance	25	25000 ppb	DOI:10.1016/j.biosx.2019.100016	2019[11]
Nonanal	Chemical resistance	25	1000 ppb	DOI:10.1016/j.cej.2019.123104	2020[12]
Nonanal	Absorbance	100	125 ppb	DOI:10.1109/JSEN.2021.3055264	2021[13]
Trans-2-hexenal	Chemical resistance	25	170 ppb	DOI:10.1016/j.matt.2021.06.009	2021[14]

Nonanal	Colorimetric	25	4500 ppb	DOI:10.1016/j.microc.2021.106988	2022[15]
Trans-2-hexenal	Colorimetric	20	8000 ppb	DOI:10.1021/acsami.4c09402	2024[16]
Trans-2-hexenal	Chemical resistance	100	17.40 ppb	DOI:10.1016/j.foodchem.2025.142845	2025[17]
Trans-2-nonenal	Chemical resistance	25	0.70 ppb	This work	2025

Section S9. Selectivity of the sensor

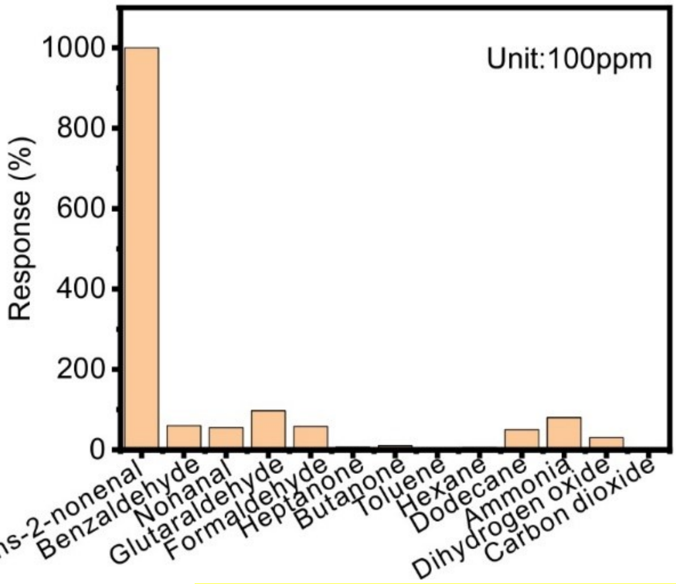

Figure S10. Selectivity of the sensor. The term response signal refers to the resistance value.

Section S10. Effect of humidity on sensor

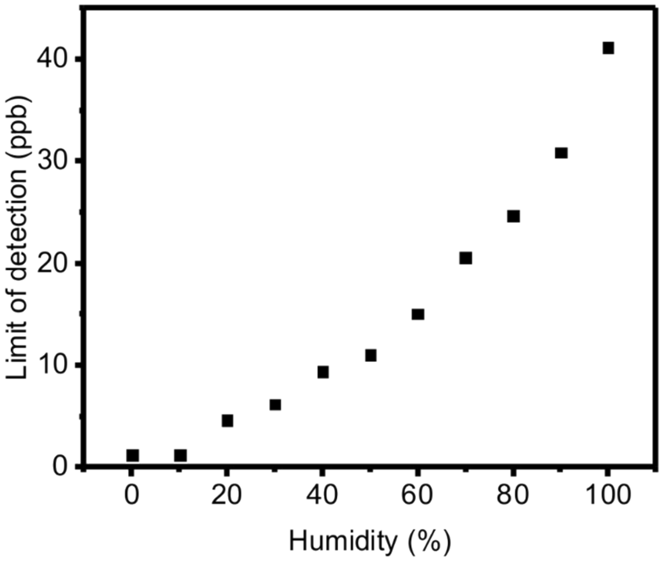

Figure S11. Effect of humidity on the limit of detection in CdSe@CdS-MBAu sensor.

Section S11. Schematic diagram of the printed circuit board module for the sensor

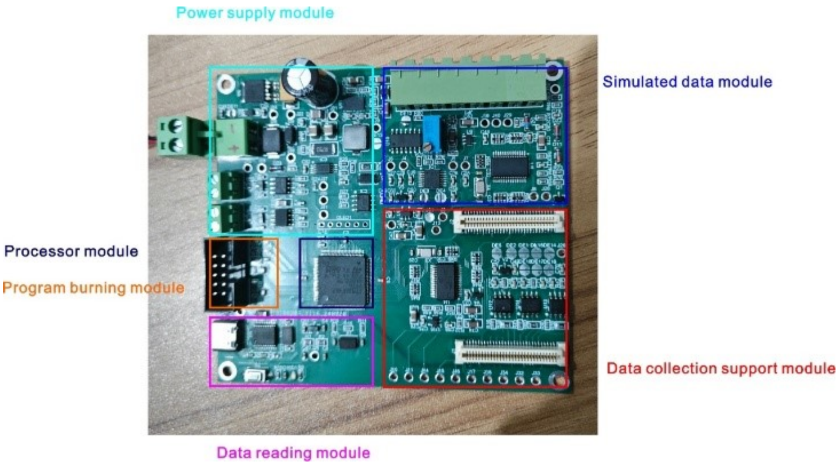

Figure S12. The designed compact circuit board for sensor portable devices.

Section S12. Scree plot of PCA

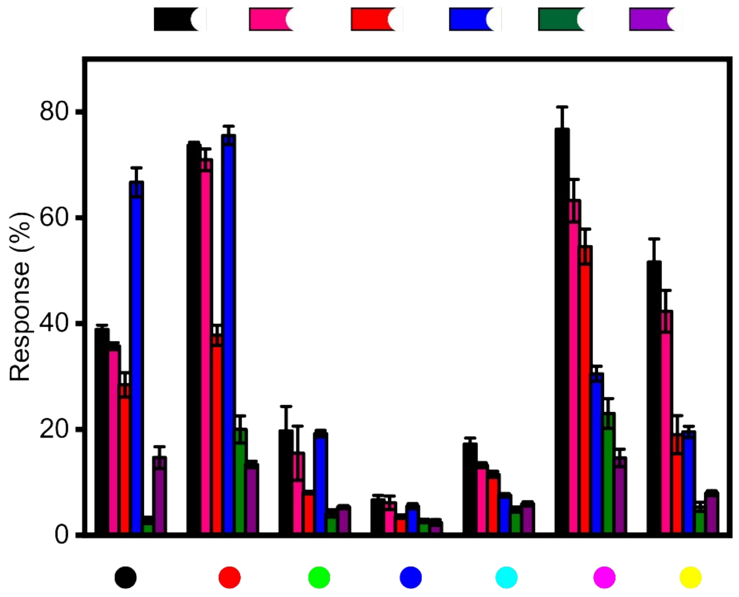

Figure S13. Responses of six channel chemoresistive sensors upon exposure to trans-2-nonenal analogs at 1 ppm plus the N_2 control. The term response signal refers to the resistance value.

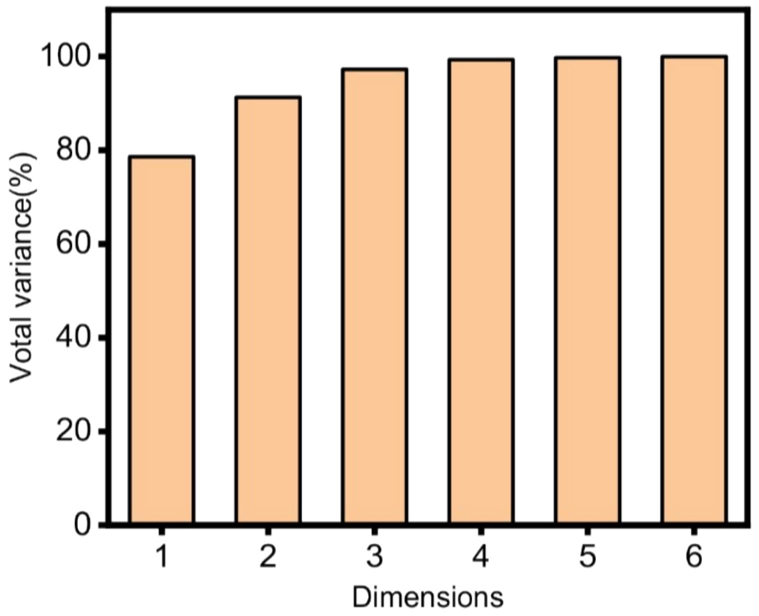

Figure S14. Scree plot of PCA of seven different aldehydes, with three principal components contributing a total variance of 97.2%.

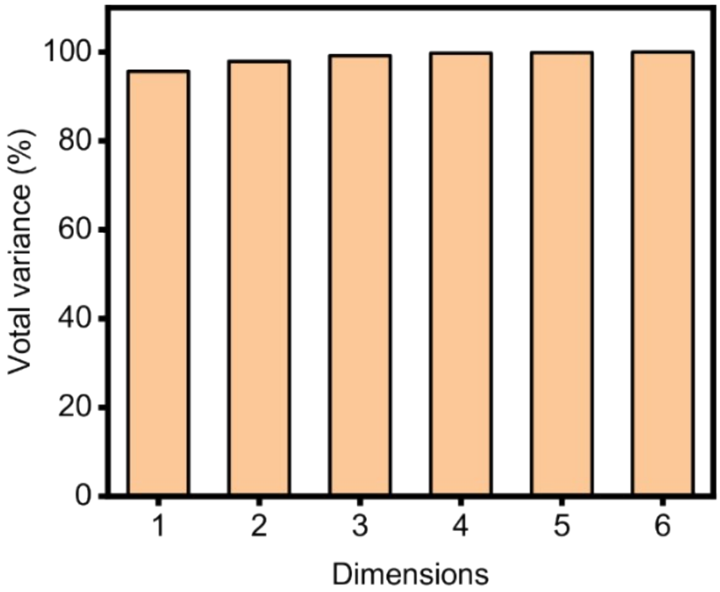

Figure S15. Scree plot of PCA of exhaled breath between simulated healthy and NSCLC patients, where the contribution rate of the two principal components to the total variance exceeds 97.7%.

References

1. S. Yang and K. Han, Effects of Solvent Dielectric Constant and Viscosity on Two Rotational Relaxation Paths of Excited 9-(Dicyanovinyl) Julolidine, *The Journal of Physical Chemistry A*, 2016, **120**, 4961-4965.
2. J. Hu, Y. Zou, Y. Deng, H.-J. Li, H. Xu, D. Wang, L. Wu, Y. Deng and G. Li, Recent advances in non-ionic surfactant templated synthesis of porous metal oxide semiconductors for gas sensing applications, *Progress in Materials Science*, 2025, **150**, 101409.
3. I. H. M. van Stokkum, D. S. Larsen and R. van Grondelle, Global and target analysis of time-resolved spectra, *Biochimica et Biophysica Acta (BBA) - Bioenergetics*, 2004, **1657**, 82-104.
4. K. Hagedorn, W. Li, Q. Liang, S. Dilger, M. Noebels, M. R. Wagner, J. S. Reparaz, A. Dollinger, J. Schmedt auf der Günne, T. Dekorsy, L. Schmidt-Mende and S. Polarz, Catalytically Doped Semiconductors for Chemical Gas Sensing: Aerogel-Like Aluminum-Containing Zinc Oxide Materials Prepared in the Gas Phase, 2016, **26**, 3424-3437.
5. R. Wang, K. Lan, Z. Chen, X. Zhang, C.-T. Hung, W. Zhang, C. Wang, S. Wang, A. Chen, W. Li, X. Xu and D. Zhao, Janus Mesoporous Sensor Devices for Simultaneous Multivariable Gases Detection, *Matter*, 2019, **1**, 1274-1284.
6. T. Itoh, T. Nakashima, T. Akamatsu, N. Izu and W. Shin, Nonanal gas sensing properties of platinum, palladium, and gold-loaded tin oxide VOCs sensors, *Sensors and Actuators B: Chemical*, 2013, **187**, 135-141.
7. Z. Gan and J. Wang, Portable hydrogel kit based on Michael addition reaction for (E)-2-hexenal gas detection, *Journal of Colloid and Interface Science*, 2024, **673**, 258-266.
8. Y. Masuda, T. Itoh, W. Shin and K. Kato, SnO2 Nanosheet/Nanoparticle Detector for the Sensing of 1-Nonanal Gas Produced by Lung Cancer, *Scientific Reports*, 2015, **5**.
9. Y. Masuda, K. Kato, M. Kida and J. Otsuka, Selective nonanal molecular recognition with SnO_2 nanosheets for lung cancer sensor, *International Journal of Applied Ceramic Technology*, 2019, **16**, 1807-1811.
10. Z. Li, R. Paul, T. Ba Tis, A. C. Saville, J. C. Hansel, T. Yu, J. B. Ristaino and Q. Wei, Non-invasive plant disease diagnostics enabled by smartphone-based fingerprinting of leaf volatiles, *Nature Plants*, 2019, **5**, 856-866.
11. B. Liu, Y. Huang, K. W. L. Kam, W.-F. Cheung, N. Zhao and B. Zheng, Functionalized graphene-based chemiresistive electronic nose for discrimination of disease-related volatile organic compounds, *Biosensors and Bioelectronics: X*, 2019, **1**, 100016.
12. A. Daneshkhah, S. Vij, A. P. Siegel and M. Agarwal, Polyetherimide/carbon black composite sensors demonstrate selective detection of medium-chain aldehydes including nonanal, *Chemical Engineering Journal*, 2020, **383**, 123104.
13. M. Tsujiguchi, T. Aitoku, H. Takase and Y. Y. Maruo, Nonanal Sensor Fabrication Using Aldol Condensation Reaction Inside Alkali-Resistant Porous Glass, *IEEE Sensors Journal*, 2021, **21**, 8868-8877.
14. Z. Li, Y. Liu, O. Hossain, R. Paul, S. Yao, S. Wu, J. B. Ristaino, Y. Zhu and Q. Wei, Real-time monitoring of plant stresses via chemiresistive profiling of leaf volatiles by a wearable sensor, *Matter*, 2021, **4**, 2553-2570.
15. A. Jahangiri-Manesh, M. Mousazadeh, M. Nikkhah, S. Abbasian, A. Moshaii, M. J. Masroor and P. Norouzi, Molecularly imprinted polymer-based chemiresistive sensor for detection of nonanal as a cancer related biomarker, *Microchemical Journal*, 2022, **173**, 106988.
16. M. Zhao, H. Lu, Z. You, H. Chen, X. Wang, Y. Zhang and Y. Wang, Olfactory Visualization Sensing Array Made with CelluMOFs to Predict Fruit Ripeness Using Deep Learning, *ACS Applied Materials & Interfaces*, 2024, **16**, 56623-56633.

17. X. She, H. Tian, Y. Lei, L. Yang, S. Zhao, J. Wu, P. Wang, J. Li and Y. Zhang, A novel electronic nose for sensing (E)-2-hexenal based on Mn-MOF nanonets with NADPH-like activity, *Food Chemistry*, 2025, **471**, 142845.
